# Recurrent Urinary Tract Infection Caused by Multidrug‐Resistant *Klebsiella pneumoniae*: A Case Report

**DOI:** 10.1155/crdi/1851069

**Published:** 2026-06-25

**Authors:** Adriano Souza Santos Monteiro, Vívian Santos Galvão, Camila Maria Piñeiro Silva, Soraia Machado Cordeiro, Joice Neves Reis

**Affiliations:** ^1^ Postgraduate Course in Biotechnology in Health and Investigative Medicine, Gonçalo Moniz Institute, Oswaldo Cruz Foundation, Salvador, Bahia, Brazil, fiocruz.br; ^2^ Postgraduate Program in Pharmacy, Faculty of Pharmacy, Federal University of Bahia, Salvador, Bahia, Brazil, ufba.br; ^3^ Faculty of Pharmacy, Federal University of Bahia, Salvador, Bahia, Brazil, ufba.br

**Keywords:** antimicrobial resistance, case report, *Klebsiella pneumoniae*, recurrent urinary tract infection

## Abstract

*Klebsiella pneumoniae* is the second most common cause of urinary tract infection (UTI) in most populations; however, few cases of recurrent UTI (rUTI) have been described. In this report, we described a patient who experienced 18 episodes of community‐acquired rUTI caused by extended‐spectrum β‐lactamase (ESBL)–producing and multidrug‐resistant (MDR) *K. pneumoniae* over an 8‐year period. All isolates were ESBL producers and MDR, with variable resistance profiles. Molecular analysis of 10 isolates also revealed variations in β‐lactamase genes; however, all isolates shared the same capsular serotype (K17) and virulence gene profile. PFGE analysis identified three closely related pulsotypes, and all isolates belonged to ST101, supporting relapse events during recurrent infection. Particular attention should be paid to empirical treatment of UTI due to the high resistance rates observed in *K. pneumoniae*, even in community‐acquired infections.

## 1. Introduction

Urinary tract infections (UTI), a condition affecting over 150 million people annually worldwide [1], is often caused by *K. pneumoniae*, the second leading causative agent in most populations [2]. The impact of UTI is further exacerbated by the emergence of extended‐spectrum β‐lactamase (ESBL)–producing *K. pneumoniae*, which confers resistance to third‐ and fourth‐generation cephalosporins and monobactams, leading to therapeutic failures with severe consequences for the patient [3–5].

Recurrent UTI (rUTI), a particularly challenging aspect of UTI, is defined as two or more episodes of UTI within 6 months or three or more UTIs within 12 months [6]. About 50% of women with UTI experience rUTI episodes within 1 year, and ∼25% experience recurrence within 6 months [7–9]. rUTI can occur due to reinfection or relapses [10]. However, studies are inconclusive, and few have confirmed the type of rUTI by molecular typing methods. This study aims to fill this gap by characterizing *K. pneumoniae* isolates obtained from a patient who experienced 18 episodes of community‐acquired rUTI over an 8‐year period.

## 2. Case Presentation

**FIGURE 1:**
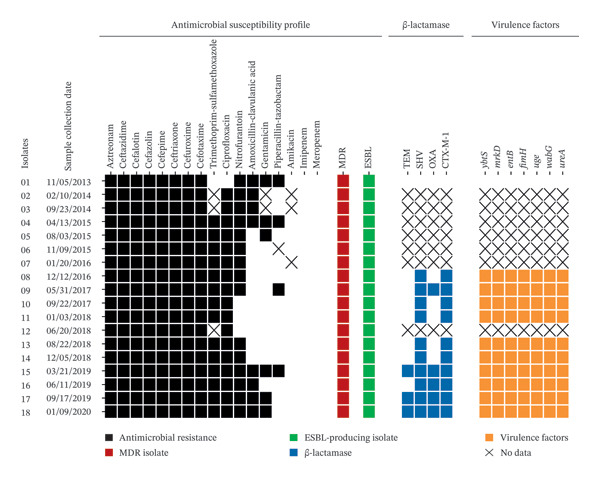
Antimicrobial resistance, β‐lactamase, and virulence profiles of 18 *Klebsiella pneumoniae* isolates recovered from a patient with community‐acquired recurrent urinary tract infection in Salvador, Brazil, between 2013 and 2020. The figure highlights the long‐term persistence of ESBL‐producing multidrug‐resistant isolates and the progressive acquisition of resistance determinants over time. Note: ESBL, extended‐spectrum β‐lactamase; MDR, multidrug‐resistant.

In November 2013, a 63‐year‐old widow with comorbidities such as diabetes mellitus, hypertension, rheumatoid arthritis, and anemia visited a university laboratory in Salvador, Brazil, for a urine culture. The patient reported UTI symptoms such as dysuria, urinary urgency, vaginal itching, and fever and met the criteria for community‐acquired infection [11]. The initial urine culture yielded positive results (≥ 100,000 CFU/mL) for ESBL‐producing and multidrug‐resistant (MDR) [12] *K*. *pneumoniae*. In February and September 2014, the patient returned to the laboratory with two other positive urine cultures for the same bacteria, which was also isolated in 2015 (April, August, and November), 2016 (January and December), 2017 (May and November), 2018 (January, June, August, and December), 2019 (March, June, and September), and in January 2020. In total, the patient experienced 18 episodes of rUTI (Figure 1).

Antimicrobial susceptibility testing was performed using the disk diffusion method [13], and results showed that all *K. pneumoniae* isolates were resistant to cephalosporins (100%, 18/18), aztreonam (100%, 17/17), and sulfamethoxazole/trimethoprim (100%, 14/14). Ninety‐four percent (17/18) of the isolates were resistant to ciprofloxacin, 83% (15/18) were resistant to nitrofurantoin, 44% (8/18) were resistant to amoxicillin/clavulanic acid, 38% (6/16) were resistant to gentamicin, and 24% (4/17) were resistant to piperacillin/tazobactam. Furthermore, all *K. pneumoniae* isolates were ESBL producers, MDR, and susceptible to carbapenems and amikacin (Figure 1).

The detection of β‐lactamase genes by PCR [14, 15] showed that bla_SHV_ and bla_CTX-M-1_ were identified in all isolates. Additionally, 50% (5/10) of the isolates showed bla_OXA-1-like_, and 30% (3/10) had bla_TEM_. Virulence genes for capsule (*uge* and *wabG*), fimbriae (*fimH* and *mrkD*), siderophores (*entB* and *ybtS*), and urease production (*ureA*) were detected in all isolates by PCR [16–20]. Furthermore, all isolates were classified as classical strains [18] and belonged to the K17 capsular serotype [21] (Figure 1).

**FIGURE 2:**
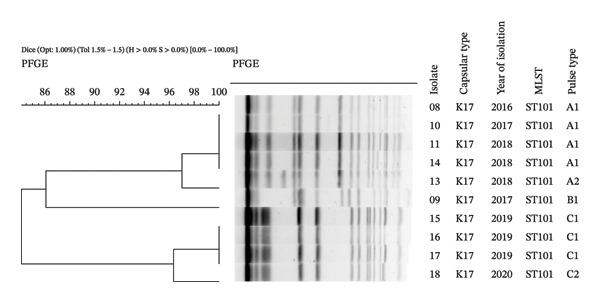
PFGE dendrogram of 10 *Klebsiella pneumoniae* isolates recovered from recurrent urinary tract infection episodes in a patient from Salvador, Brazil. The analysis demonstrated long‐term clonal persistence of ST101 K17 isolates, despite the presence of closely related pulsotypes over different years of infection. Note: MLST, multilocus sequence typing; PFGE, pulsed‐field gel electrophoresis.

*K. pneumoniae* isolates were also analyzed by PFGE using *Xba*I‐digested DNA, as previously described [5]. The analysis identified three closely related pulsotypes, all sharing > 80% similarity: A (five isolates), B (one isolate), and C (four isolates) (Figure 2). Interestingly, Patterns A and B occurred in rUTIs from 2016 to 2018, while Pattern C occurred in rUTIs from 2019 to 2020. MLST (https://bigsdb.pasteur.fr/klebsiella/) revealed that all isolates belonged to ST101.

## 3. Discussion

This study describes episodes of rUTI caused by ESBL‐producing and MDR *K. pneumoniae*, where we observed relapse events with genetically related subpopulations. In this study, the patient had some of the risk factors for developing UTI, such as old age and diabetes mellitus [22]. Diabetes is also a risk factor for rUTI [23] and appears to be associated with rUTI caused by *K. pneumoniae* [24]. The patient also had rheumatoid arthritis, an autoimmune disease treated with immunosuppressants, which can increase susceptibility to infections. For example, the annual incidence of UTI in elderly patients with rheumatoid arthritis was 2.09%, which was higher than the incidence in those without the disease (0.91%) [25].

Over an 8‐year period, 18 episodes of rUTI were identified as being caused by ESBL‐producing and MDR *K. pneumoniae* isolates, with closely related clonal profiles observed among the 10 isolates analyzed by PFGE. In cases of UTI in community patients, empirical antibiotic therapy is typically the initial approach [26]. However, treatment options for *K. pneumoniae* infections are often limited due to the high rates of antimicrobial resistance [27]. Amoxicillin/clavulanic acid would be effective in 56% of the rUTI episodes in the patient described in this study. However, the remaining therapeutic options could only be administered in a hospital setting.

A study found that UTIs caused by ESBL‐producing Enterobacterales have higher recurrence rates compared to those caused by non‐ESBL–producing strains [28]. Several other studies have also reported higher recurrence rates among patients with UTI caused by ESBL‐producing Enterobacterales [29–31]. It is worth noting that in this study, CTX‐M was the β‐lactamase responsible for the ESBL phenotype in all isolates, which is expected as this enzyme predominates in ESBL‐producing Enterobacterales worldwide [32].

A study conducted in Taiwan also identified relapsed episodes of rUTI caused by *K. pneumoniae*: 17 episodes in seven patients, with one patient experiencing five episodes and six patients experiencing two episodes each. In contrast to our findings, the antimicrobial susceptibility profile of the recurrent isolates remained consistent with that of the original isolate in all cases [24]. Therefore, we hypothesize that subpopulations of isolates causing rUTI in this study may have acquired different resistance genes over time. The identification of closely related pulsotypes over multiple years, all belonging to the same ST101 lineage, strongly supports relapse events rather than independent reinfections, highlighting the importance of molecular typing methods in the investigation of rUTI. *K. pneumoniae* ST101 is a high‐risk MDR clone that has been increasingly reported in different infections worldwide [33].

All isolates analyzed in this report were identical in terms of the virulence profile. The *K. pneumoniae* isolates were classified as classical strains that are less overtly pathogenic but represent most isolates associated with infections, especially among elderly patients with comorbidities [18, 34]. Presumably, classical strains were expected since these isolates are more prone to acquiring antimicrobial resistance genes than hypervirulent ones [34]. The isolates in our case were also similar in their capsular serotype, and the virulence genes identified were either intrinsic or present in integrative and conjugative elements that also integrate into the chromosome (e.g., *ybtS* gene) [35, 36].

This study has some limitations. First, this report was primarily developed from a microbiological and longitudinal laboratory surveillance perspective. Therefore, detailed information regarding clinical management, therapeutic interventions, and long‐term outcomes was not fully available, mainly because the isolates were obtained from a community laboratory with limited access to the patient’s complete medical records. In addition, the patient has not returned to the laboratory for new urine cultures since the last episode in 2020. Second, the loss of eight isolates made it impossible to complete the antimicrobial susceptibility profile and molecular characterization of all isolates. Finally, although PFGE and MLST demonstrated high discriminatory power, whole genome sequencing could provide more detailed information regarding the genetic relatedness and longitudinal evolution of isolates during rUTI. Despite these limitations, to our knowledge, this is the first report describing rUTI caused by MDR ESBL‐producing *K. pneumoniae* over such a prolonged follow‐up period (8 years).

## 4. Conclusion

We concluded that the rUTI episodes described in this report were most likely related to relapse events caused by closely related ESBL‐producing and MDR *K. pneumoniae* ST101 subpopulations, as demonstrated by molecular typing methods. Finding this resistance phenotype in isolates from community‐acquired UTIs is concerning, as it makes appropriate antimicrobial therapy even more challenging. Therefore, empirical treatment, often used to treat UTI, carries a high risk of severe clinical consequences for the patient. It is crucial to identify and report the existence of *K. pneumoniae* with this resistance phenotype in rUTI and explore alternative therapeutic strategies to manage the patient’s condition effectively.

## Author Contributions

Adriano Souza Santos Monteiro: formal analysis, investigation, data curation, writing–original draft, and writing–review and editing. Vívian Santos Galvão: investigation, data curation, and writing–review and editing. Camila Maria Piñeiro Silva: investigation, data curation, and writing–review and editing. Soraia Machado Cordeiro: conceptualization, methodology, formal analysis, writing–review and editing, supervision, and project administration. Joice Neves Reis: methodology, formal analysis, writing–review and editing, resources, and supervision.

## Funding

This work was supported by the Fundação de Amparo à Pesquisa do Estado da Bahia (FAPESB, grant number PP‐SUS0014/2018) and the Coordenação de Aperfeiçoamento de Pessoal de Nível Superior (CAPES–financial code 001). Adriano Souza Santos Monteiro acknowledges FAPESB for the Ph.D. scholarships, and Joice Neves Reis acknowledges financial support from the National Council for Scientific and Technological Development (CNPq, grant number 309564/2022).

## Disclosure

This work was previously presented at the 58th Congress of the Brazilian Society of Tropical Medicine (MEDTROP 2023), Salvador, Brazil. The funding agencies had no role in the study design, data collection and analysis, or the decision to publish this report.

## Ethics Statement

This study was approved by the local Research Ethics Committee under authorization number 79527717.1.0000.8035 and was conducted with the patient’s authorization by means of a signed informed consent form.

## Conflicts of Interest

The authors declare no conflicts of interest.

## Data Availability

The data that support the findings of this study are available on request from the corresponding author.
